# Influence of carbon free gaseous ammonia induction on combustion, performance and emissions in an agricultural diesel engine operated on dual fuel mode

**DOI:** 10.1038/s41598-025-32413-z

**Published:** 2025-12-13

**Authors:** Naseem Khayum, Syed Suraya, Yerumbu Nandakishora, Jakeer Hussain Shaik, Debabrata Barik, Milon Selvam Dennison, Ayyar Dinesh, Saravanan Rajendran

**Affiliations:** 1 Department of Mechanical Engineering, Dayananda Sagar University, Bangalore, 562112 Karnataka India; 2 Department of Electrical Engineering, College of Engineering, King Khalid University, Abha, Saudi Arabia; 3 Department of Mechanical Engineering, Presidency University, Bangalore, India; 4 Department of Computer Science & Engineering, KITS AKSHAR Institute of Technology, Guntur, Andhra Pradesh India; 5 Department of Mechanical Engineering, Karpagam Academy of Higher Education, Coimbatore, 641021 India; 6 Centre for Energy and Environment, Karpagam Academy of Higher Education, Coimbatore, 641021 India; 7 Department of Mechanical Engineering, Kampala International University, Western Campus, P.O. Box 71, Ishaka-Bushenyi, Uganda; 8 Department of Chemistry, K. Ramakrishnan College of Engineering (Autonomous), Affiliated to Anna University, Samayapuram, Trichy, 621112 Tamil Nadu India; 9 Instituto de Alta Investigación, Universidad de Tarapacá, Arica, 1000000 Chile

**Keywords:** Ammonia, Combustion, Performance, Diesel engine, Jatropha methyl ester, Energy science and technology, Engineering

## Abstract

The transition towards cleaner fuels is very important due to its potential to reduce greenhouse emissions and favor the decarbonized engine operation. Recently, Ammonia (NH_3_) has emerged as a promising carbon-free energy carrier and alternative fuel, which can replace traditional fossil fuels. This study aims to showcase the procedure of using NH_3_ as a primary fuel with 20% Jatropha biodiesel and 80% diesel, designated as JME20 as a pilot fuel in dual-fuel mode. Hence, a single-cylinder DI diesel engine was retrofitted to induct NH_3_ into the intake manifold, whereas JME20 is being injected and sprayed into the engine cylinder to initiate the combustion. NH_3_ was inducted at different proportions, such as 8, 10, 12, and 16 lpm, which are designated as DFX, DFX1, DFX2, and DFX3, respectively. Experimentation was carried out at different engine loading conditions, such as 0%, 25%, 50%, 75% and 100%. At each load, the corresponding engine characteristics, namely combustion, performance, and emissions, were measured, compared with standard diesel fuel and given in the paper. Results reveal that a maximum of 24.3% NH_3_ was replaced for the DFX3 test fuel at full load. Increasing NH_3_ share will extend the delay period from 10.9°CA to 12.6°CA for 12 lpm (DFX2); and lengthen the combustion duration (CD) from 43.3°CA to 48.3°CA for the same fuel at full load. Moreover, the peak cylinder pressure increased from 55.4 bar to 58.6 bar, also a 6.7% rise in maximum heat release rate and 4.2% improvement in BTE at 12 lpm. A percentage increase in CO & HC emissions by about 54.3% and 51.8% respectively, than diesel at full load. These findings confirm that 12 lpm (DFX2) is the most balanced and optimum condition, validating NH_3_-JME20 as a promising strategy as a sustainable pathway for agricultural engines.

## Introduction

The ever-growing global demand for energy, together with the faster depletion of fossil fuel reserves, has escalated the search for a sustainable and clean source of energy. Traditional fossil fuels are not only limited in nature but also significantly contribute to greenhouse gas (GHG) emissions, and other harmful pollutants, which leads to deteriorate the climate and quality of air. As a result, the agricultural and transportation sectors, which rely heavily on internal combustion (IC) engines, are under tremendous pressure to transition towards cleaner and renewable fuel alternatives^1–4^.

In recent decades, several liquid and gaseous alternative fuels like biodiesel, ethanol, biogas, hydrogen, and natural gas have received wide research attention for their utilization in IC engines. Though these fuels have recorded mixed levels of achievements in enhancing combustion efficiency and reducing some emissions, issues like storage, cost, volumetric energy density, and necessity for engine modifications still restrain their large-scale use. Among gaseous fuels, hydrogen has received wide attention owing to its relatively high flame speed and zero carbon emission^5–8^. However, the difficulties like low volumetric energy density and storage, necessitate the exploration of other gaseous energy carriers^9^.

NH_3_ has recently emerged as a promising carbon-free energy carrier and alternative fuel. High hydrogen content and the ability to release no carbon dioxide (CO_2_) emissions upon combustion are the most unique characteristics of NH_3_ to use as a fuel for IC engines. In addition, NH_3_ can be synthesized using renewable energy via the Haber-Bosch process, making it as an attractive option for future low-carbon energy systems. However, its high auto-ignition temperature, low flame speed, and narrow flammability limits pose significant challenges for its use in conventional engines. To overcome these challenges, strategies such as dual-fuel operation, pilot ignition have been proposed^10,11^.

Recent research has highlighted the growing potential of using ammonia (NH₃) as an alternative fuel in internal combustion engines. One key investigation^12^ explored the performance of NH₃ in spark-ignition (SI) engines and demonstrated that while the peak cylinder pressure (PCP) decreased slightly due to NH_3_’s relatively low flame speed, the overall engine power improved notably. The study also indicated that direct ammonia injection effectively reduced carbon monoxide (CO) emissions but led to higher levels of nitrogen oxides (NOx) and hydrocarbons (HC). These outcomes confirm NH_3_’s suitability for SI engines, provided that the injection timing and pressure are carefully optimized to control emissions.

In compression-ignition (CI) engines, NH₃ shows even greater promise, largely due to its high energy content and near-zero carbon emissions. Several investigations^13^ have examined dual-fuel strategies, where NH_3_ is introduced through the intake while diesel or biodiesel is used as the pilot fuel for ignition. In one such study, Niki et al.^14^ observed that increasing the ammonia fraction in the intake resulted in a corresponding rise in NH_3_ emissions. Similarly, Yousefi et al.^15^ reported a slight drop in thermal efficiency with higher NH₃ substitution but also noted a reduction in NOx emissions, particularly when advanced pilot injection strategies were employed to lower greenhouse gas output. Nadimi et al.^16^ further showed that up to 84.1% of the engine’s total energy input could be replaced by NH_3_, leading to a substantial improvement in thermal efficiency and a marked decline in carbon-based emissions, although they emphasized the need for strategies to mitigate increased NOx levels. Complementing these findings, experiments by Kaiyuan Cai et al.^17^ revealed that incorporating NH_3_ into diesel combustion prolongs both ignition delay and overall combustion duration.

A significant contribution in this field was made by Liang Zheng and co-workers^18^, who analyzed the performance behavior of a diesel engine operated with varying ammonia (NH_3_) blending ratios. Their findings indicated that at higher NH_3_ shares (around 60%), the engine achieved a peak thermal efficiency of approximately 43.5%, reflecting enhanced combustion quality and lower carbon-based emissions. However, they also emphasized that determining the most suitable NH_3_ proportion is essential to achieve an effective compromise between NH_3_ utilization and engine performance. In a related investigation, Liu and Liu^19^ focused on identifying the optimal NH_3_ share in a dual-fuel configuration using NH_3_ and diesel.

Further insight into emission behavior from blended fuels was provided by Reiter and Kong^20^, who examined the co-combustion of diesel and NH_3_. In their experiments, vaporized NH_3_ was introduced through the intake manifold, while diesel was injected into the combustion chamber to initiate ignition. The study employed a constant engine power output while varying the NH_3_-diesel energy fractions. The most efficient operating condition was observed at diesel/NH_3_ energy ratios between 40 and 60% and 60–40%. Compared to conventional diesel-only operation, dual-fuel combustion resulted in lower hydrocarbon (HC) and carbon monoxide (CO) emissions. Moreover, when NH₃ contributed less than 40% of the total energy, NO_X_ formation decreased significantly due to the lower combustion temperature. Conversely, when NH_3_ became the dominant fuel, the nitrogen content contributed to a marked increase in NOx emissions. The use of NH_3_ also suppressed soot formation because of its carbon-free nature. Cylinder pressure analysis revealed that increasing NH_3_ content reduced peak pressure and extended the ignition delay period. Overall, the dual-fuel strategy showed lower CO_2_ and CO emissions than conventional diesel, although high NH_3_ concentrations (above 60%) were associated with a sharp rise in NOx emissions.

A new combustion strategy for utilizing ammonia in compression ignition (CI) engines was proposed by Lee and Song^21^ with the objective of lowering NO emissions. Through a series of parametric studies, they validated and analyzed the behavior of an ammonia–diesel dual-fuel engine under different operating conditions. Their work highlighted how variations in the ammonia injection quantity and start of injection (SOI) timing directly influenced NOx formation. It was found that, for fixed ammonia and diesel quantities, NOx emissions were more sensitive to SOI than to engine load, with measured NOx levels dropping from 8500 ppm to 3040 ppm when SOI was optimized. In a related study, Yousefi et al.^22^ examined the combined effects of ammonia energy fraction and diesel injection timing. They observed a 58.8% reduction in NOx emissions when the ammonia energy share increased from 0% to 40%, though this was accompanied by higher N_2_O emissions a potent greenhouse gas.

Several investigations have consistently shown that increasing the NH_3_ substitution fraction reduces peak in-cylinder pressure and shifts the heat release rate (HRR) peak later in the cycle due to slower combustion kinetics. For example, Nadimi et al.^16^ reported that increasing ammonia substitution to 84% lowered peak cylinder pressure by several bar and lengthened ignition delay by more than 3°CA. These effects were partially countered by advancing pilot injection timing to around 16°CA. Similarly, Reiter and Kong^23^ found that using 80% ammonia (by energy) significantly delayed HRR and prolonged combustion duration, highlighting the inherently low reactivity of ammonia. Ma et al.^24^ reported analogous behavior in marine diesel engines, whereas Niki et al.^25^ observed higher combustion temperatures and reduced N_2_O emissions during ammonia fumigation though they cautioned that improper dosing could lead to NH_3_ slip.

Injection strategies and pilot fuel proportions have been identified as key parameters for improving overall performance. Sivasubramanian et al.^26^ demonstrated that applying a 45% biodiesel pilot injection advanced HRR by approximately 20% toward TDC, shortened ignition delay by 23%, and increased brake thermal efficiency (BTE) to 36.22%, representing a 12.33% gain compared to single injection. Brake specific energy consumption (BSEC) was also reduced by 19.31%. Similar findings were reported by Nadimi et al.^27^, who achieved over 33% reductions in HC, CO, and smoke emissions, though accompanied by a 36% rise in NOx levels. Furthermore, Jayabal et al.^28^ observed that moderate ammonia enrichment (6 L min⁻¹) enhanced BTE from 31.1% to 34.8%, largely due to improved mixing and more stable combustion.

Additive-assisted strategies have also been investigated to enhance engine performance with ammonia-based dual fueling. For instance, Pugazhendhi et al.^29^ observed that introducing 75 ppm of CeO_2_ nanoparticles into a castor biodiesel–ammonia blend reduced the combustion duration by approximately 3°CA and advanced CA50. This modification led to a 22.2% rise in thermal efficiency for B10 blends and a 26% decrease in brake specific fuel consumption (BSFC), accompanied by a slight increase in NOx emissions of about 4.3%. The catalytic behavior of CeO_2_ promotes improved oxidation, thereby counterbalancing the efficiency penalties typically associated with high ammonia substitution.

Across multiple studies, emission trends consistently indicate lower CO, HC, smoke opacity, and CO₂ levels when ammonia is used as a co-fuel. This is largely attributed to the absence of carbon in ammonia. For example, Sivasubramanian et al.^26^ reported reductions of 34% in HC, 39% in CO, and 34% in smoke emissions. However, a recurring issue in these investigations is the increase in NOx emissions, which in some cases reached up to 36%, as highlighted by Jamrozik et al. and Reiter, and Kong^13,23^. To address this trade-off, advanced control strategies such as optimized injection timing, split injection techniques, and exhaust aftertreatment have been recommended.

A closer examination of the literature on ammonia-fueled diesel engines provides further insights. Numerous researchers^30^ have employed either diesel or biodiesel as a pilot fuel with ammonia as the main energy source. Findings indicate that cylinder pressure tends to decrease due to ammonia’s lower combustion reactivity and broader flammability range, resulting in a prolonged ignition delay^31,32^. During this extended delay, a larger quantity of fuel accumulates and undergoes improved vaporization, which eventually increases peak cylinder pressure^31–33^. Studies also show an increase in CO and HC emissions under these conditions^34^. While some reports noted a significant reduction in NO emissions^35^, others observed a rise in NO levels^36^. In contrast, smoke emissions generally exhibited a declining trend with ammonia induction^37^.

### Research gap and objective of this investigation

Although several studies have explored ammonia as a supplementary fuel in SI and CI engines, the majority have focused on automotive applications using diesel as a pilot fuel. Very limited research has addressed NH_3_ induction in agricultural engines, which are crucial for rural energy security and operate under distinct load profiles. Furthermore, the potential of biodiesel as a sustainable pilot fuel in NH_3_-assisted dual-fuel engines has not been fully explored. As per the author’s understanding, Jatropha biodiesel (B20) as a pilot fuel was not explored in NH_3_-fueled diesel engines. To address this gap, the present study investigates the effect of NH_3_ induction at varying flow rates (8–16 lpm) on the combustion, performance, and emission characteristics of a 4-stroke, DI agricultural diesel engine using JME20 as the pilot fuel. The results are compared against baseline diesel operation to assess the viability of NH_3_ as a renewable gaseous fuel for agricultural applications.

## Methodology and details of the test rig

### Test fuels

Diesel was purchased from a retail station of Indian Oil Pvt. Ltd, located near the premises of our campus. On the other hand, Jatropha Methyl Ester (JME) was purchased from Biofuzel International Limited, Madhya Pradesh, India. The properties of JME were measured and compared with the standard diesel fuel, presented in Table 1. The primary fuel (NH_3_) was also purchased from Sri Varadayini Enterprises, Visakhapatnam, India. JME20 was chosen as the pilot fuel because numerous studies have proved that B20 is the optimum blend that compromises combustion quality and engine compatibility without requiring major modifications.

**Table 1:** Comparison of test fuels.

Test Method	Units	Diesel	JME	JME20	NH_3_ (gas)	ASTM Method adopted
Cetane number	-	50	55	51	-	D4737
Kinematic viscosity @313 K	mm^2^/s	2.2	5.4	2.84	-	D445
Lower heating value	kJ/kg	43.4	39.4	42.6	18.6	D3338
Flash Point	K	329	429	355	−33℃	D93
Density @288 K	kg/m^3^	820	878	832	0.73	D1298
Carbon	%	86.2	77.1	84.0	0	D3178
Hydrogen	%	13.2	11.81	12.9	17.6	D3178
Nitrogen	%	Nil	0.119	0.02	82.4	D3179
Sulphur	%	0.3	0.001	0.24	Nil	D3177
Oxygen by difference	%	Nil	10.97	2.19	Nil	E385

### Experimentation facility

**Fig. 1:**
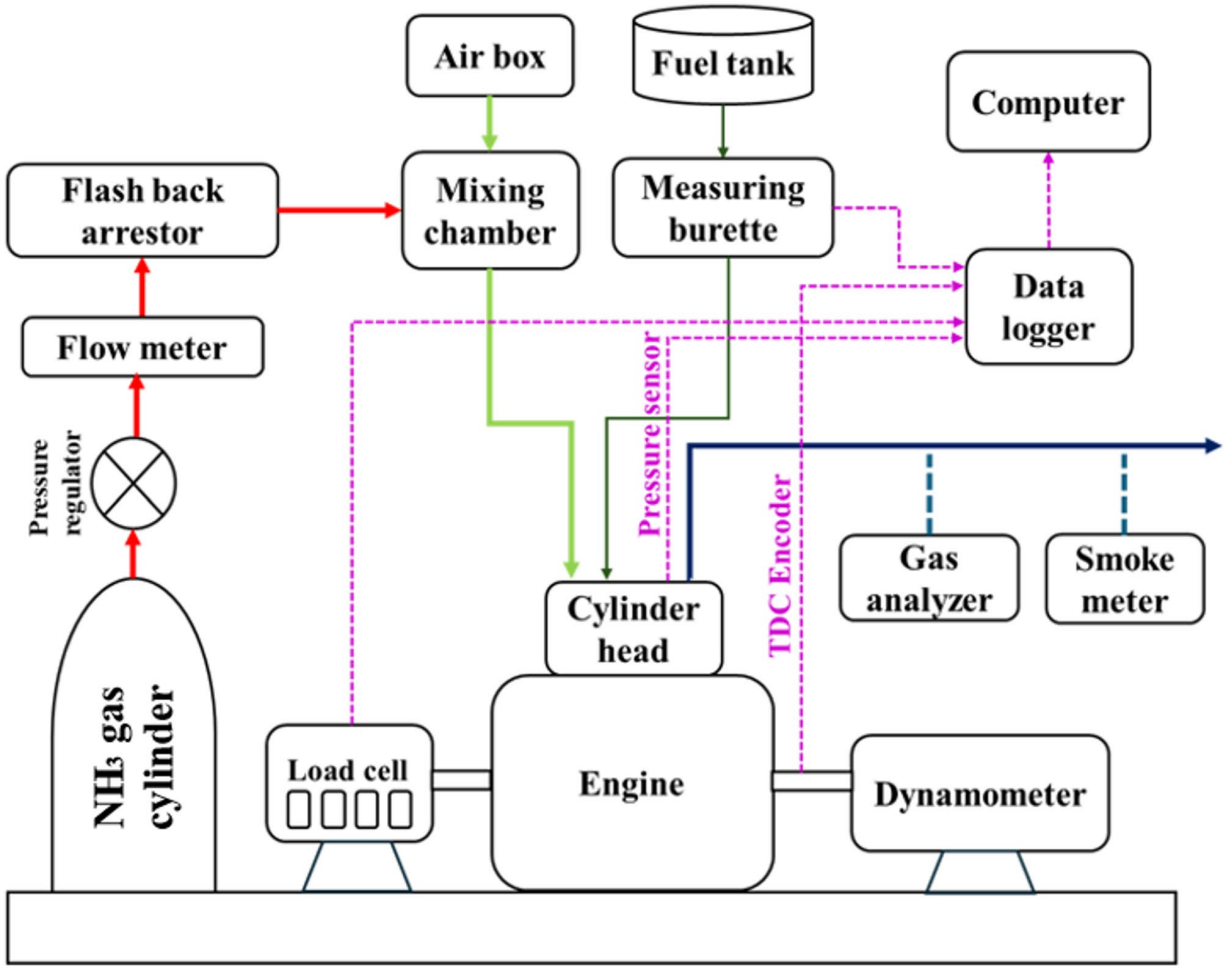
Schematic layout of test rig.

The experimental investigations were performed on a 4-stroke, single-cylinder, naturally aspirated, DI diesel engine that was operated at a constant speed of 1500 rpm. The engine was suitably modified to function in DFO, retrofitted to induct gaseous NH_3_ through the intake port while injecting JME20 as pilot fuel. An external NH_3_ supply line also connected to the intake port through a calibrated control valve. A multi hole (venturi based) gas mixing unit was also mounted on the intake port to ensure proper mixing. No alteration was made to the injector, fuel pump, injection timing. This retrofit allows simultaneously NH_3_ + air during the intake stroke of the engine, enabling DFO. A schematic arrangement of the test facility is illustrated in Fig. 1, and the key specifications of the engine are summarized in Table 2. The complete test rig was procured from M/s. Legion Brothers, Bengaluru, India. Loading was applied and monitored by means of an electrical alternator coupled to the crankshaft through a load cell.

The air flow rate into the engine was quantified using a U-tube manometer in combination with a sharp-edged orifice plate. Fuel consumption was determined by a vertical burette of 30 cm³ capacity, which was fitted with two optical sensors at the upper and lower ends; the effective measurement volume was 20 cm³ between the sensors.

Temperatures at critical points, namely the intake air, exhaust gases, and ammonia line, were monitored with K-type thermocouples. Engine speed was recorded by a non-contact type sensor positioned adjacent to the flywheel. In-cylinder pressure data were acquired at an interval of 0.5° crank angle (°CA) using a piezoelectric transducer (Kistler, Model 5395 A) mounted on the cylinder head. A high-resolution crank angle encoder was employed to detect crank position and the top dead center (TDC). At each operating point, approximately 1050 data points of pressure and volume were recorded per cycle, and the heat release rate (HRR) was obtained by averaging over 20 successive cycles. The output signals from the encoder and pressure sensor were routed through a charge amplifier and subsequently fed into a computer-based data acquisition system (DAS) for analysis and storage.

Exhaust gas emissions were analyzed in accordance with ASTM D6522. During steady operation, the exhaust stream was drawn through a sampling probe, passed through filters, and dehumidified using a condensation trap. The dried sample was then analyzed with a nondispersive infrared (NDIR) analyzer for CO, CO₂, and HC, while NO concentrations were measured with an electrochemical detector. Smoke opacity in the exhaust was determined using an AVL 437 C diesel smoke meter.

**Table 2:** Technical specifications of the engine setup.

Engine type	4-stroke, single cylinder, air cooled
Make	Kirloskar TAF1
Test fuel	Diesel, JME + NH_3_
Bore x Stroke (mm)	87.5 × 110
Clearance (mm)	1.1–1.2
Rated Speed (rpm)	1500
Brake power (kW)	4.4
Swept volume (cm^3^)	661
Compression ratio	17.5:1
Fuel injection	Direct
Standard injection pressure (bar)	200
Standard injection timing (^o^ CA)	23
Injector nozzle	3 hole
Dynamometer	Electrical loading
Combustion chamber	Hemispherical type
IVO (^o^CA)	4.5 bTDC
IVC (^o^CA)	35.5 aBDC
EVO (^o^CA)	35.5 aBDC
EVC (^o^CA)	4.5 aTDC
Starting	Crank starting

### Ammonia handling & leakage prevention measures

To prevent NH_3_ leakage during the experimentation, the NH_3_ cylinder was equipped with a dual-stage pressure regulator with a check valve. Chemical-resistant PTFE gas lines with compression fittings were employed, and all the joints were tested for leaks before each test using an NH_3_-detection spray. The test lab was also equipped with a mechanical ventilation system and an NH_3_ warning sensor. During operation, pressure stability was observed in the induction line, while the purging of the system with fresh air was done before shutdown of the NH_3_ supply.

### Details of the instruments & uncertainty analysis

The assessment of uncertainty analysis is crucial for measuring the accuracy of an instrument, and was carried out using the formulae as given in^38^. Table 3 portrays the list of uncertainties in the instruments used for this study.

5 $${{\mathrm{U}}_{\mathrm{R}}}=({\mathrm{A}}_{{\mathrm{R}}}^{{\mathrm{2}}}{\mathrm{+B}}_{{\mathrm{R}}}^{{\mathrm{2}}}{\mathrm{)}}$$

Where, U_R_ refers to the uncertainty of the estimated parameter at 95% confidence level. While A_R_ indicates the systematic and B_R_ refers to random uncertainties.

6 $$\frac{{{A_R}}}{R}={\left( {{{\sum\limits_{{i=1}}^{n} {\left( {\frac{1}{R}\frac{{\partial R}}{{\partial {X_i}}}{A_i}} \right)} }^2}} \right)^{0.5}}$$

7 $$\frac{{{B_R}}}{R}={\left( {{{\sum\limits_{{i=1}}^{n} {\left( {\frac{1}{R}\frac{{\partial R}}{{\partial {X_i}}}{B_i}} \right)} }^2}} \right)^{0.5}}$$

In the above relation, R is the estimated parameter that relies on the variable Xi. The symbols A_i_ and A_R_ represent the measurement level and uncertainty in R, respectively.

**Table 3:** Instruments used in this study.

Instrument used	Range	Accuracy	Measurement	Percentage of uncertainty
Pressure transducer	0–110	± 0.1	In-cylinder pressure, bar	± 0.15
Load cell	250–6000	± 10	Load on the engine with aid of dynamometer, W	± 0.2
Data acquisition system	64	± 0.1	Converts analog to digital, bit	± 0.001
Temperature indicator	0–900	± 1	Measures the EGT, inducted air, biogas, ^o^C	± 0.15
Charge amplifier	-	± 1	Converts charge to voltage	± 0.1
Speed sensor	0–10000	± 10	Speed, rpm	± 1
Crank angle encoder	0–720	± 0.6	Crank angle, ^o^CA	± 0.01
Burette	1–30	± 0.2	Fuel consumption, cm^3^	± 0.5
Air flow meter	0.5–50	± 0.1	Air consumption, m^3^/min	± 0.5
Gas flow meter	0.1–25	± 0.1	NH_3_ gas consumption, m^3^/min	± 0.02
Exhaust gas analyser	0–5000	± 50	NO, ppm	± 1
	0–20000	± 10	HC, ppm	± 0.5
	0–10	± 0.03	CO, %	± 0.03
Smoke metre	0–100	± 1	Smoke density, %	± 1

By performing the repeatability of the experiments, the uncertainty for the parameters EGT, BSFC, BTE, HC, CO, NO, and smoke density was calculated as,

$${{\mathrm{[(EGT}}{{\mathrm{)}}^{\mathrm{2}}}{\text{+ (BSFC}}{{\mathrm{)}}^{\mathrm{2}}}{\text{+ (BTE}}{{\mathrm{)}}^{\mathrm{2}}}{\text{+ (HC}}{{\mathrm{)}}^{\mathrm{2}}}{\text{+ (CO}}{{\mathrm{)}}^{\mathrm{2}}}{\text{+ (NO}}{{\mathrm{)}}^{\mathrm{2}}}{\text{+ (Smoke}}{{\mathrm{)}}^{\mathrm{2}}}{\mathrm{]}}^{{\mathrm{0}}{\mathrm{.5}}}}$$

$${\text{= [(0}}{\mathrm{.15}}{{\mathrm{)}}^{\mathrm{2}}}{\text{+ (0}}{\mathrm{.5}}{{\mathrm{)}}^{\mathrm{2}}}{\text{+ (0}}{\mathrm{.5}}{{\mathrm{)}}^{\mathrm{2}}}{\text{+ (0}}{\mathrm{.5}}{{\mathrm{)}}^{\mathrm{2}}}{\text{+ (0}}{\mathrm{.03}}{{\mathrm{)}}^{\mathrm{2}}}{\text{+ (1}}{{\mathrm{)}}^{\mathrm{2}}}{\text{+ (1}}{{\mathrm{)}}^{\mathrm{2}}}{{\mathrm{]}}^{{\mathrm{0}}{\mathrm{.5}}}}{{= \pm 1}}{\text{.66\% }}$$

### Ammonia energy share

In DFO, the energy share of gaseous fuel is an important parameter when analysing the impact of premixed combustion. In order to produce some power, both the gaseous fuel or primary fuel (NH_3_) and the pilot fuel (JME20) should contribute energy. It is also noted from the figure that the pilot fuel consumption varies with the load, whereas the primary fuel remains unchanged with the change in load. It is also understood that the energy share is a strong function of rate of fuel consumption and calorific value. The below formulae shown the calculation of energy share, where mpilot fuel, CVpilot fuel and $\:{\mathrm{m}}_{{NH}_{3}}$, $\:\mathrm{C}{\mathrm{V}}_{{NH}_{3}}$ represent the mass of fuel consumption and calorific value of pilot and primary fuels, respectively. The energy ratio of NH_3_ at different engine loads is given in Table 4 for the test fuels such as DFX, DFX1, DFX2, and DFX3.

The energy share of $\:{NH}_{3}$ was calculated using the following formulae^39^;

1 $$\:Energy\:share\:of\:{NH}_{3}=\frac{Energy\:equivalent\:of\:{NH}_{3}}{Energy\:equivalent\:of\:(NH3+pilot\:fuel)}\times\:100$$

Where;

2 $$\:\mathrm{E}\mathrm{n}\mathrm{e}\mathrm{r}\mathrm{g}\mathrm{y}\:\mathrm{e}\mathrm{q}\mathrm{u}\mathrm{i}\mathrm{v}\mathrm{a}\mathrm{l}\mathrm{e}\mathrm{n}\mathrm{t}\:\mathrm{o}\mathrm{f}\:{NH}_{3}=\:\frac{{\mathrm{m}}_{{NH}_{3}}\times\:\mathrm{C}{\mathrm{V}}_{{NH}_{3}}}{3600}$$

3 $$\:\mathrm{En}\mathrm{e}\mathrm{r}\mathrm{g}\mathrm{y}\:\mathrm{e}\mathrm{q}\mathrm{u}\mathrm{i}\mathrm{v}\mathrm{a}\mathrm{l}\mathrm{e}\mathrm{n}\mathrm{t}\:\mathrm{o}\mathrm{f}\:\mathrm{p}\mathrm{i}\mathrm{l}\mathrm{o}\mathrm{t}\:\mathrm{f}\mathrm{u}\mathrm{e}\mathrm{l}=\:\frac{{\mathrm{m}}_{\mathrm{p}\mathrm{i}\mathrm{l}\mathrm{o}\mathrm{t}\:\mathrm{f}\mathrm{u}\mathrm{e}\mathrm{l}}\times\:\mathrm{C}{\mathrm{V}}_{\mathrm{p}\mathrm{i}\mathrm{l}\mathrm{o}\mathrm{t}\:\mathrm{f}\mathrm{u}\mathrm{e}\mathrm{l}}}{3600}$$

Also, the excess air ratio can be defined as;

4 $$\:Excess\:air\:ratio\:=\:\frac{{m}_{air}}{{\left({\left(\frac{A}{F}\right)}_{NH3}\right)}_{stoic}\times\:\:{m}_{NH3}\:+\:{\left({\left(\frac{A}{F}\right)}_{pilot\:fuel}\right)}_{pilot\:fuel}\times\:{m}_{pilot\:fuel}}$$

**Table 4:** Energy-ratio of different test fuels used in this study.

Engine load	DFX (8 lpm)	DFX1 (10 lpm)	DFX2 (12 lpm)	DFX3 (16 lpm)
No load (0%)	23.21	35.24	45.23	54.24
25%	17.23	26.28	32.36	45.26
50%	11.02	18.32	27.46	39.44
75%	8.34	14.37	21.34	31.21
Full load (100%)	6.76	12.31	18.27	24.32

**Fig. 2:**
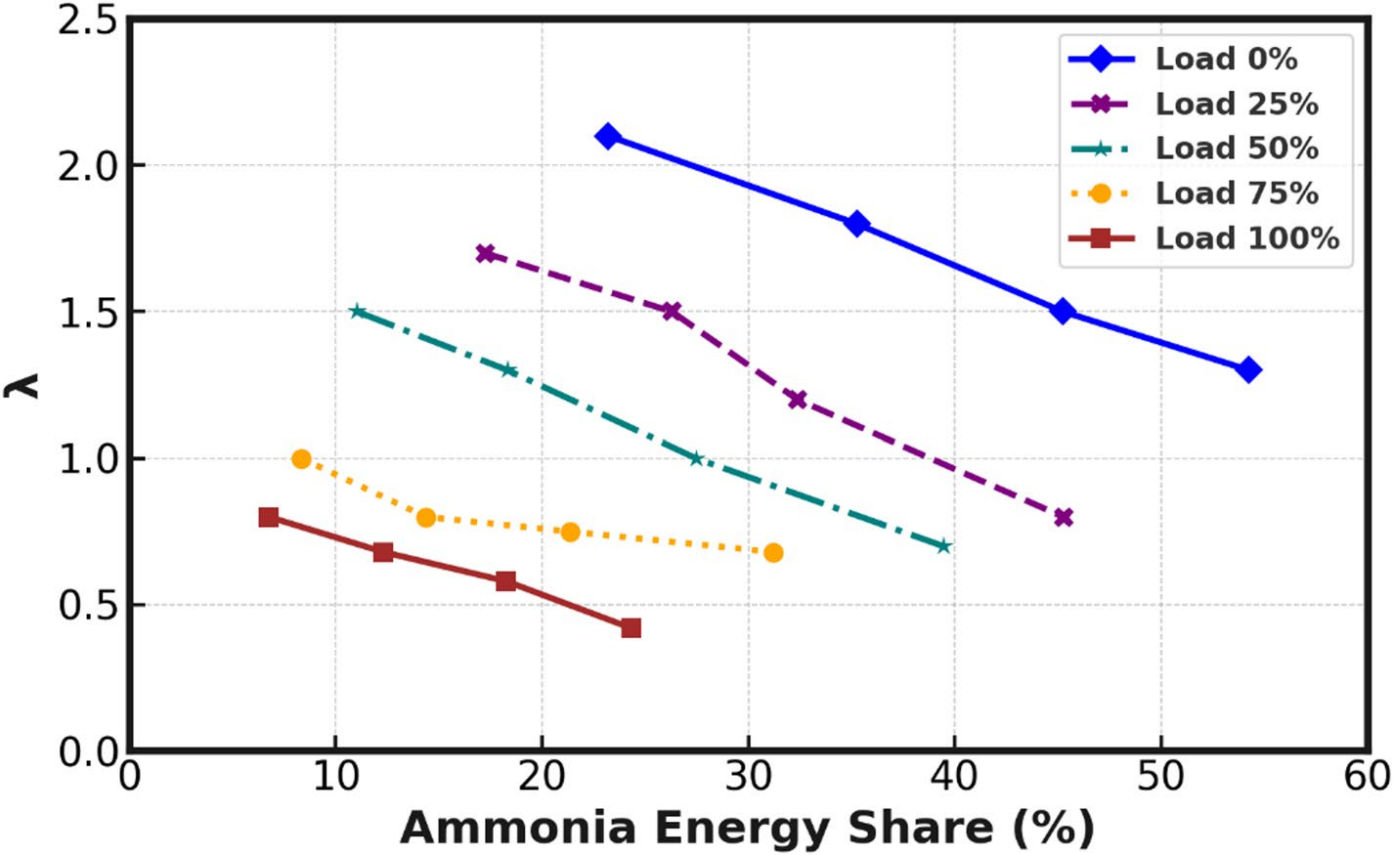
Variation of equivalence ratio with NH₃ energy share.

Figure 2 displays the relation between NH_3_ energy share and λ (lambda) at different engine loads. The energy share values of NH_3_ range from 54.6% to 6.7% (no load to full load). The energy share was high at no load and low at full load. Combustion might not be as effective, particularly at no load, which would mean the air-fuel mixture won’t burn properly, and hence more amount of fuel is required to produce the power. As the NH_3_ energy share increases, the λ value decreases, indicating a transition towards a richer air-fuel mixture. At load 0%, λ starts at its highest value, around 2.0, and decreases to 1.0 as the NH_3_ energy share approaches 50%. At load 25%, λ drops from 1.8 to approximately 1.2, and at Load 50%, it continues to decline from 1.5 to around 0.8. For load 75%, the decrease in λ becomes less pronounced, indicating a reduced sensitivity to ammonia energy share at higher loads, and at load 100%, λ reaches its lowest point, from 0.7 to 0.4, corresponding to the highest ammonia energy share. This trend highlights the effect of NH_3_ fumigation on combustion characteristics, with higher ammonia flow rates leading to a richer fuel-air mixture and optimized combustion at higher engine loads. The figure demonstrates the decreasing λ values across all loads, signifying the influence of ammonia energy share on the combustion process, particularly under varying engine load conditions. During the entire engine operation, the DFX3 shows the maximum energy share when compared to other flow rates used for this study. The energy share in % for different flow rates was found to be 6.7%, 12.3%, 18.2% and 24.3% for DFX, DFX1, DFX2, and DFX3 respectively, at full load.

## Results and discussions

### Assessment of combustion parameters

#### P-Ɵ analysis

**Fig. 3:**
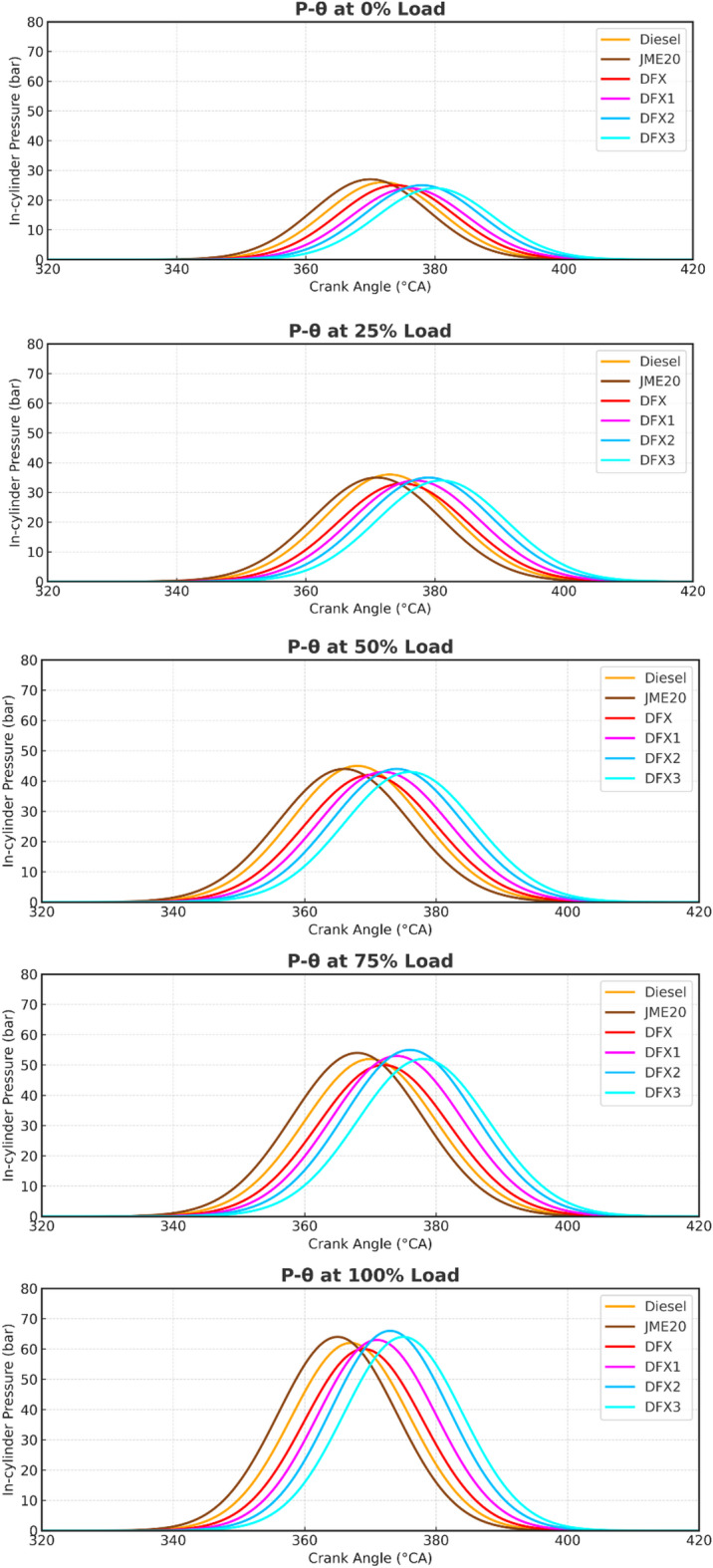
Cylinder pressure variation with respect to crank angle.

Cylinder pressure when measured as a function of crank angle (P-Ɵ curve) gives the real-time behavior of combustion inside the engine. It enables the determination of ignition delay, combustion phasing, and heat release rate (HRR) characteristics, which are very essential for evaluating engine performance. Figure 3 portrays the P-Ɵ curve for different test fuels used in this study. It is observed from the figure that diesel exhibits a higher peak pressure than the other test fuels used in this study. This might be due to the higher calorific value of diesel. It can be noted that the commencement of ignition for diesel is at 348.5°CA, whereas for B20, the ignition occurs 1.5°CA earlier. This is due to the presence of O_2_ bounded molecule in the biodiesel, which favors for earlier combustion. The PCP of diesel and B20 at full load are 62.8 bar and 61.7 bar, which occur at 10.08°CAaTDC and 7.7°CAaTDC, respectively. The lower peak value of B20 is due to the lower heating value of the fuel. In DFO, with an increase in the flow rate of ammonia, the peak pressure also increases. The lower combustion rate and slow flame speed of NH_3_ favors the ignition delay to prolong, which results in higher cylinder pressure in the premixed phase of combustion. PCP of DFX, DFX1, DFX2, and DFX3 are 61.8 bar, 55.41 bar, 56.56 bar, 58.72 bar, and 56.47 bar, occurred at 10.6°CAaTDC, 11.8°CAaTDC, 13.04°CAaTDC, and 15.42°CAaTDC, respectively, at full load.

The presence of ammonia did not significantly affect the peak pressure, but a variation in ignition delay and peak pressure shift towards the expansion process is observed. The reason stated here is in good agreement with the reason explained by Nadimi et al.^16^ in the experimentation conducted using different proportions of NH_3_ in a diesel engine.

#### HRR analysis

HRR analysis for diesel, B20, and all the dual fuel operations was calculated by using the first law of thermodynamics, which is given below^40^;

$$d{Q_{hrr}}=\left( {\left( {\frac{\gamma }{{\gamma - 1}}} \right)p\frac{{dV}}{{d\theta }}+V\left( {\frac{1}{{\gamma - 1}}} \right)\frac{{dp}}{{d\theta }}} \right)$$

From the above equation, all the right-hand terms can be easily derived with the pressure history data. The left-hand terms represent the net heat release rate in J/°CA. The HRR is a vital tool to find out the combustion duration and the delay period, which are the two basic parameters through which the combustion phenomenon can be easily identified^41^.

**Fig. 4:**
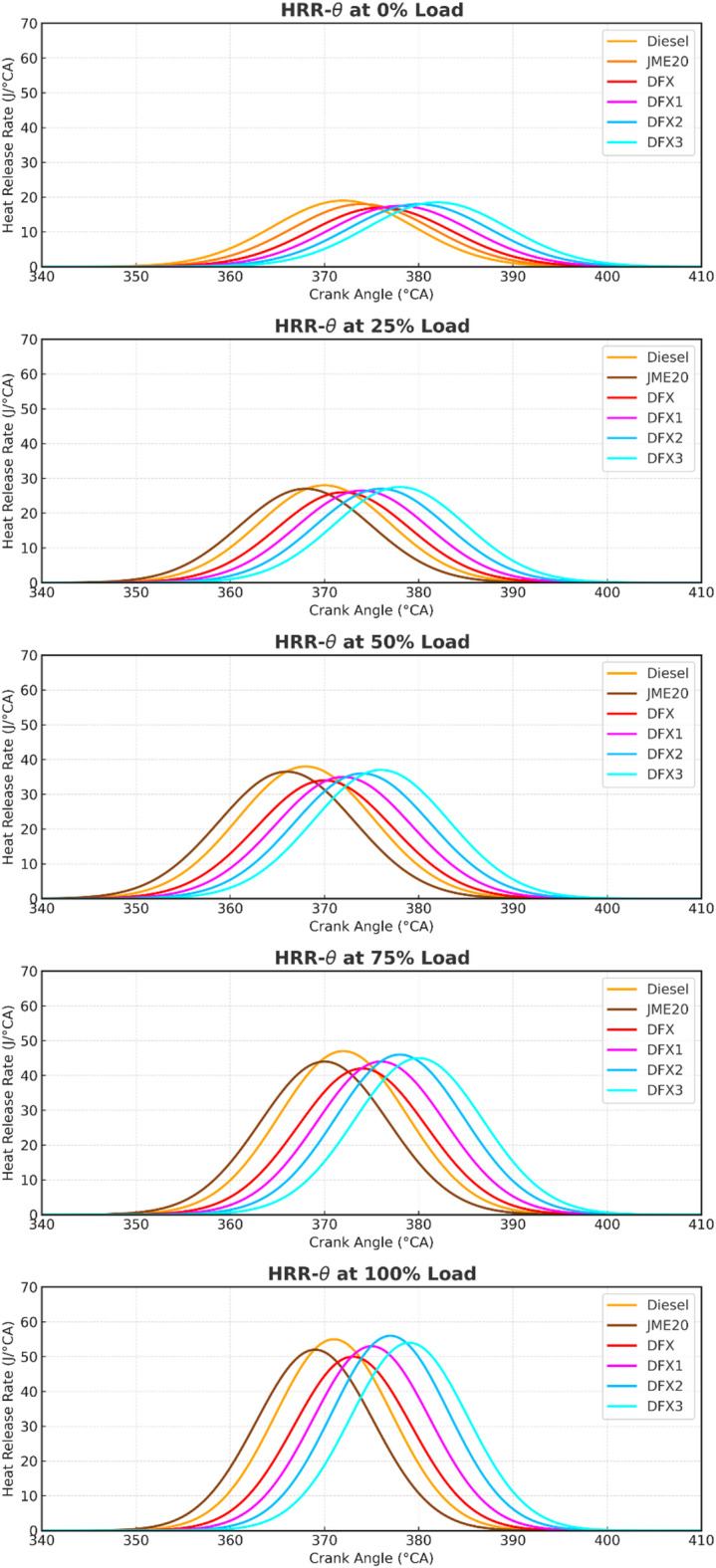
Variation of HRR with respect to crank-angle.

The HRR in the premixed phase of combustion depends on several factors like ignition delay, mixture formation, and combustion rate. Figure 4 depicts the variation of HRR with respect to crank angle for different test fuels. At full load, the HRR for diesel is 56.4 J/°CA, whereas for B20 it is 54.4 J/°CA. In single fuel operation (Diesel and B20), a sharp HRR peak is noticed, which is a characteristic of rapid premixed combustion. It is observed from the figure that diesel exhibits a higher peak due to its high volatility and calorific value. Though B20, an oxygenated fuel, shows a slightly lesser HRR peak due to its lower heating value and slower vaporization. However, the HRR curve for the ammonia/biodiesel dual fuel mode differs because of the high premixed NH_3_-air ratio. Therefore, as the NH_3_ flow rate increases from 8 to 12 lpm, a rise in the HRR peak is observed. Specifically, the peak HRR values are 51.1 J/°CA, 52.7 J/°CA, and 54.4 J/°CA for 8 lpm, 10 lpm, and 12 lpm, respectively, at full load. This increase in peak HRR corresponds to the higher amount of fuel available in the combustion chamber due to prolonged ignition delay, allowing more premixing and a more intense premixed combustion phase. Further, the combustion in dual fuel operation is retarded due to the lower combustion rate of ammonia. However, at DFX3 operation (16 lpm), the HRR peak drops back to 51.7 J/°CA. This reduction is attributed to the excessive presence of NH_3_ in the combustion chamber, which suppresses the overall combustion rate. On the other hand, the expansion pressure is slightly higher for the DFM case due to the late combustion of NH_3_.

The earlier mentioned reason was documented by Nadimi et al.^16^ in their experimentation while pointing out HRR. It is also noticed that DFX2 exhibits a higher HRR of about 3.8% than DFX, 0.8% than DFX1, and 2.4% than DFX3 at full load.

#### Ignition delay

**Fig. 5:**
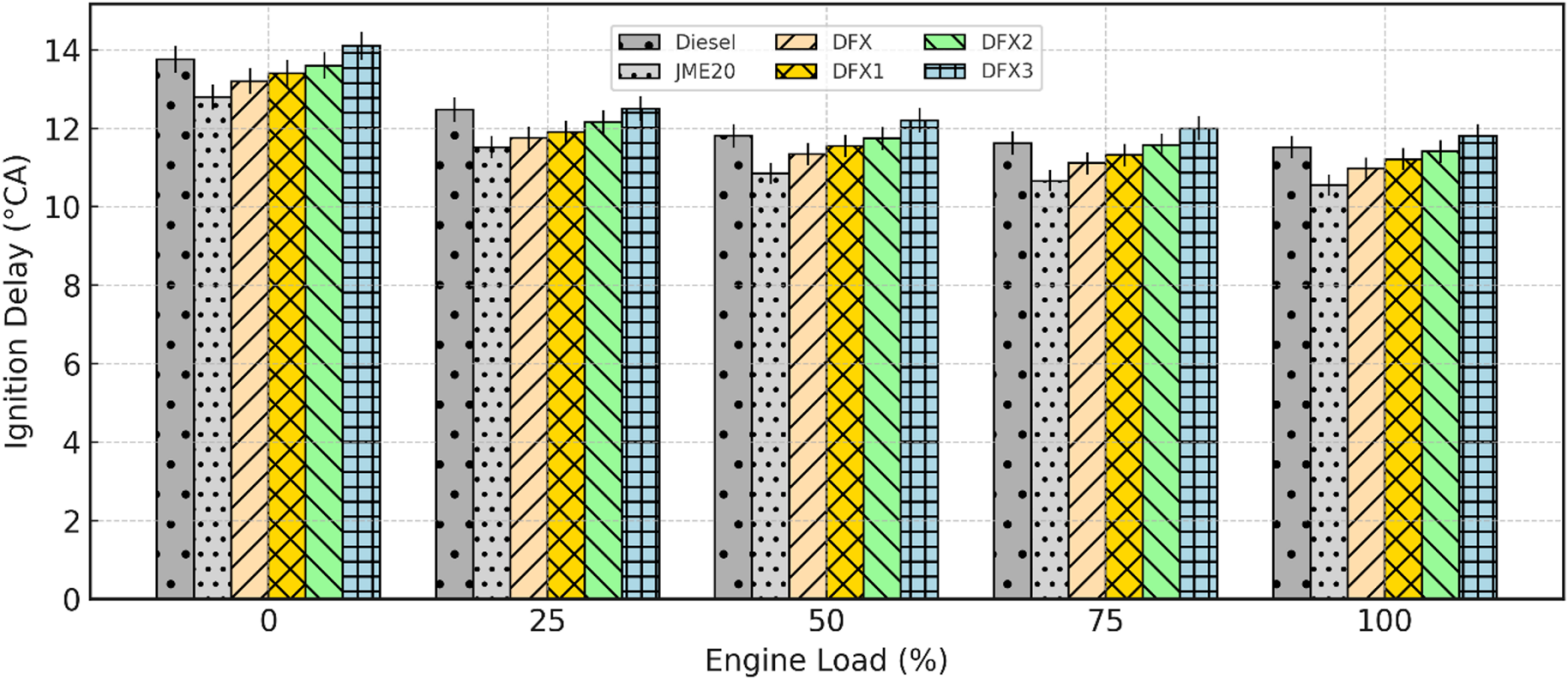
Variation of ID with respect to engine load.

The variation of ignition delay (ID) with engine load for diesel, JME20, and all DFOs is shown in Fig. 5. ID is the time or crank angle period calculated in degrees crank.

angle between the start of injection (SOI) and the start of combustion (SOC) of the mixture.

^42,43^. The factors which affect ID are fuel properties, in-cylinder pressure and temperature, air-fuel ratio and charge composition, injection timing, and pressure^42,44,45^. For all test fuels, ID decreases with increasing load due to a rise in in-cylinder temperature, which accelerates fuel-air reactions. At full load, the ID for diesel and B20 was measured as 11.5°CA and 10.5°CA, respectively. The shorter ID of B20 compared to diesel is attributed to its higher cetane number and oxygenated nature.

In DFO, ID was consistently higher than the baseline diesel operation across all loads. For example, at 50% load, the ID for DFX3 was approximately 0.7°CA longer than diesel. This extension is primarily due to the high autoignition temperature, slow flame speed, and dilution effect of NH_3_, which reduces the O_2_ concentration in the charge. Another reason might be due to the known ignition-resistant nature of NH_3_, which demands higher temperatures and longer residence time to initiate combustion. This reason was mentioned by^46^ in the experimentation carried out using NH_3_. The ID increased progressively with an increase in NH_3_ flow rates, with DFX3 showing the maximum ID across the load spectrum. At full load, ID values of DFX, DFX1, DFX2, and DFX3 are 10.9°CA, 11.2°CA, 11.4°CA, and 11.8°CA, respectively at full load.

#### Combustion duration (CD)

**Fig. 6:**
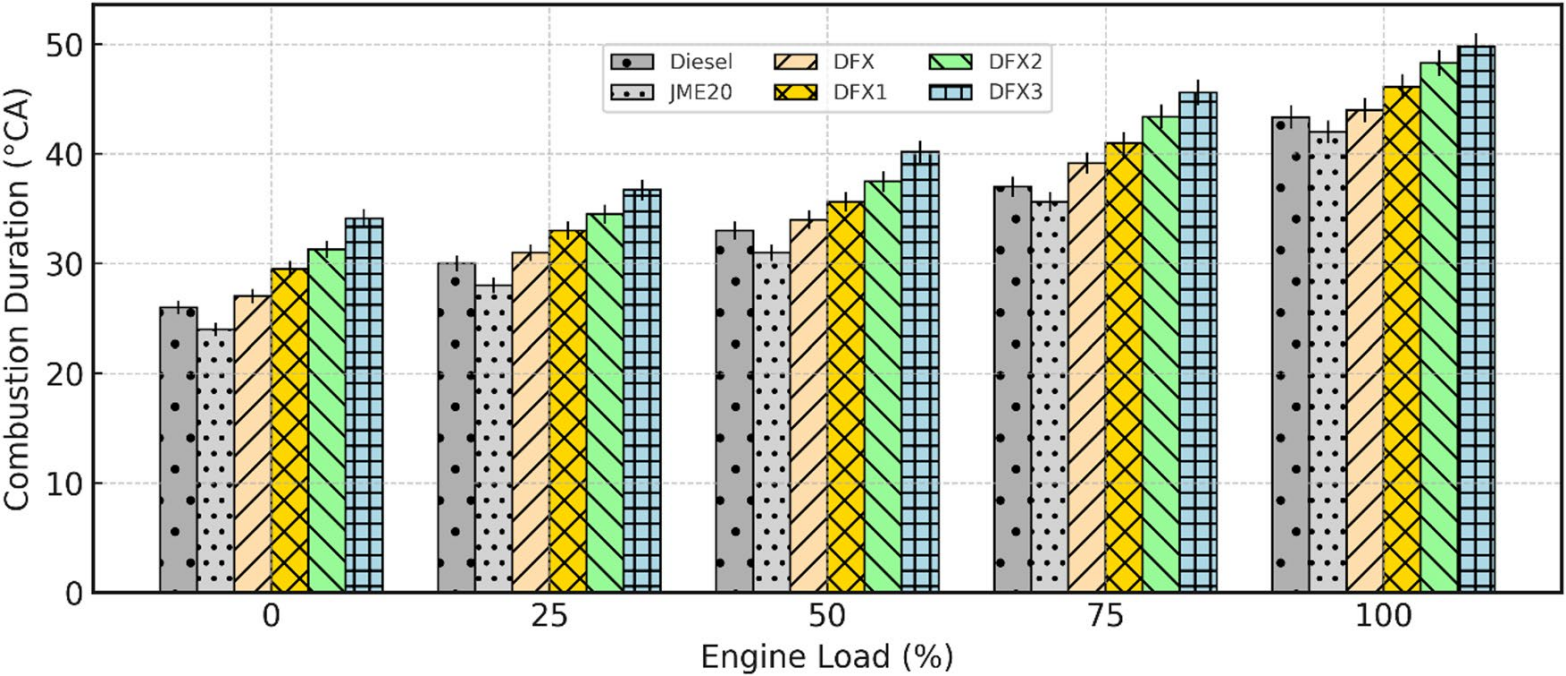
Variation of CD with respect to engine load.

Figure 6 shows the variation of combustion duration with engine load for diesel, JME20, and dual-fuel (NH_3_ + JME20) operations. CD refers to the crank angle interval between the SOC and the end of combustion (EOC). It reflects how long the fuel-air mixture takes to release most of its chemical energy and is a key parameter affecting engine performance, efficiency, and emissions. From the figure, it is observed that CD generally increases with load for all test fuels. At higher loads, more fuel is injected, leading to increased mixing and combustion phases that take a longer time to complete, thus extending the combustion duration. Among the fuels tested, JME20 exhibits the shortest CD across all load conditions. This can be attributed to its higher O_2_ content and better combustion reactivity, which support faster flame propagation and more complete combustion in a shorter period. In contrast, diesel shows slightly longer CD than JME20 due to its relatively slower burning rate and lack of inherent O_2_ content in the fuel.

In DFM, CD is significantly longer than both diesel and JME20. This is due to the presence of NH_3_, which has a low flame speed, high ignition resistance, and requires higher energy for sustained combustion. As NH_3_ concentration increases (i.e., from 8 to 16 lpm), the combustion process becomes slower and more diffused, resulting in a longer duration. The reduced O_2_ availability in the intake mixture, because NH_3_ displaces part of the intake air, also contributes to slower combustion kinetics. This reason mentioned here is aligned with the reason documented by^18^ in the experimentation carried out using NH_3_ fueled diesel engines. At full load, the CD for the test conditions of diesel, JME20, DFX (8 lpm NH_3_), DFX1 (10 lpm NH_3_), DFX2 (12 lpm NH_3_), and DFX3 (16 lpm NH_3_) are 43.3°CA, 42.1°CA, 45.2°CA, 46.1°CA, 47.2°CA, and 48.3°CA respectively. It is seen that the longest CD occurs at the highest NH_3_ flow rate (16 lpm), confirming the retarding effect of NH_3_ on flame propagation.

### Assessment of performance parameters

#### Brake thermal efficiency (BTE)

**Fig. 7:**
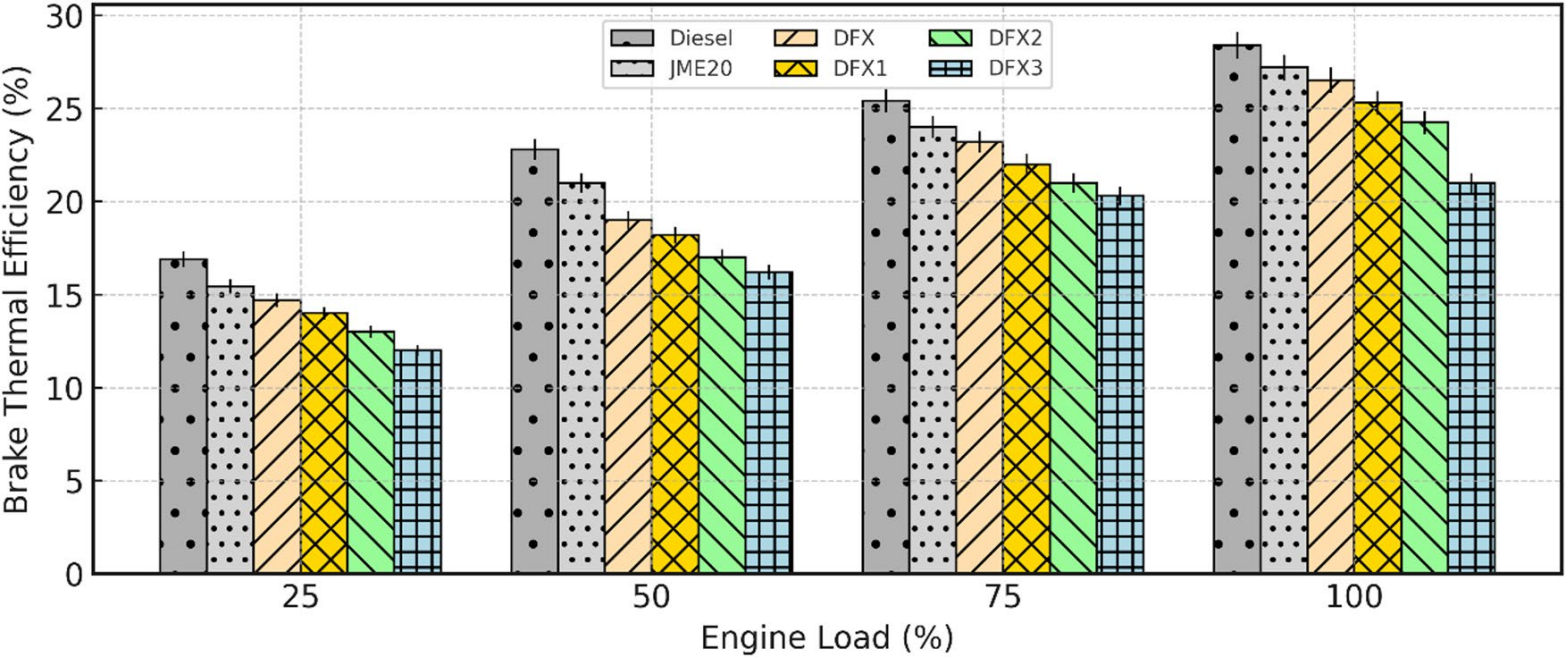
Variation of BTE with respect to engine load.

Figure 7 depicts the variation of BTE with engine load at different test fuels used in this study. It is obvious that BTE increases with the load, due to an increase in the cylinder temperature at higher loads. B20 exhibits slightly lower BTE when compared to diesel at all engine loading conditions due to higher viscosity, low calorific value, and slower evaporation^46,47^. The BTE values of sole diesel and B20 operations are about 29.8% and 27.2% at full load. In the case of DFM, BTE tends to reduce with a higher NH_3_ share at all engine loads.

For the same pilot fuel (B20), a drop in BTE is observed in the DFM of about 3.5% for DFX2 when compared to diesel operation at full load. This decrease in BTE in DFO is due to the induction of more amount of NH_3_ into the intake manifold, replacing some part of the O_2_ concentration and resulting in a decrease in fuel conversion efficiency. Another reason might be that of lower combustion speed and lower flame propagation velocity of NH_3_ lead to higher heat loss to the cylinder walls and cooling system before all the fuel is completely burned. This reduces the amount of heat available to do useful work on the piston, decreasing the BTE in DFO. These observations agree with earlier findings^46^, where NH_3_ is being substituted with biodiesel in a diesel engine. The BTE of diesel is 29.8%, whereas it is 26.5%, 25.3%, 24.2% and 21.1% for DFX, DFX1, DFX2, and DFX3, respectively, at full load. In DFM, a drop in BTE of about 6.6%, 10.9%, 14.7% and 25.7% for DFX, DFX1, DFX2, and DFX3, respectively, than that of diesel operation at full load.

#### Brake specific fuel consumption (BSFC)

**Fig. 8:**
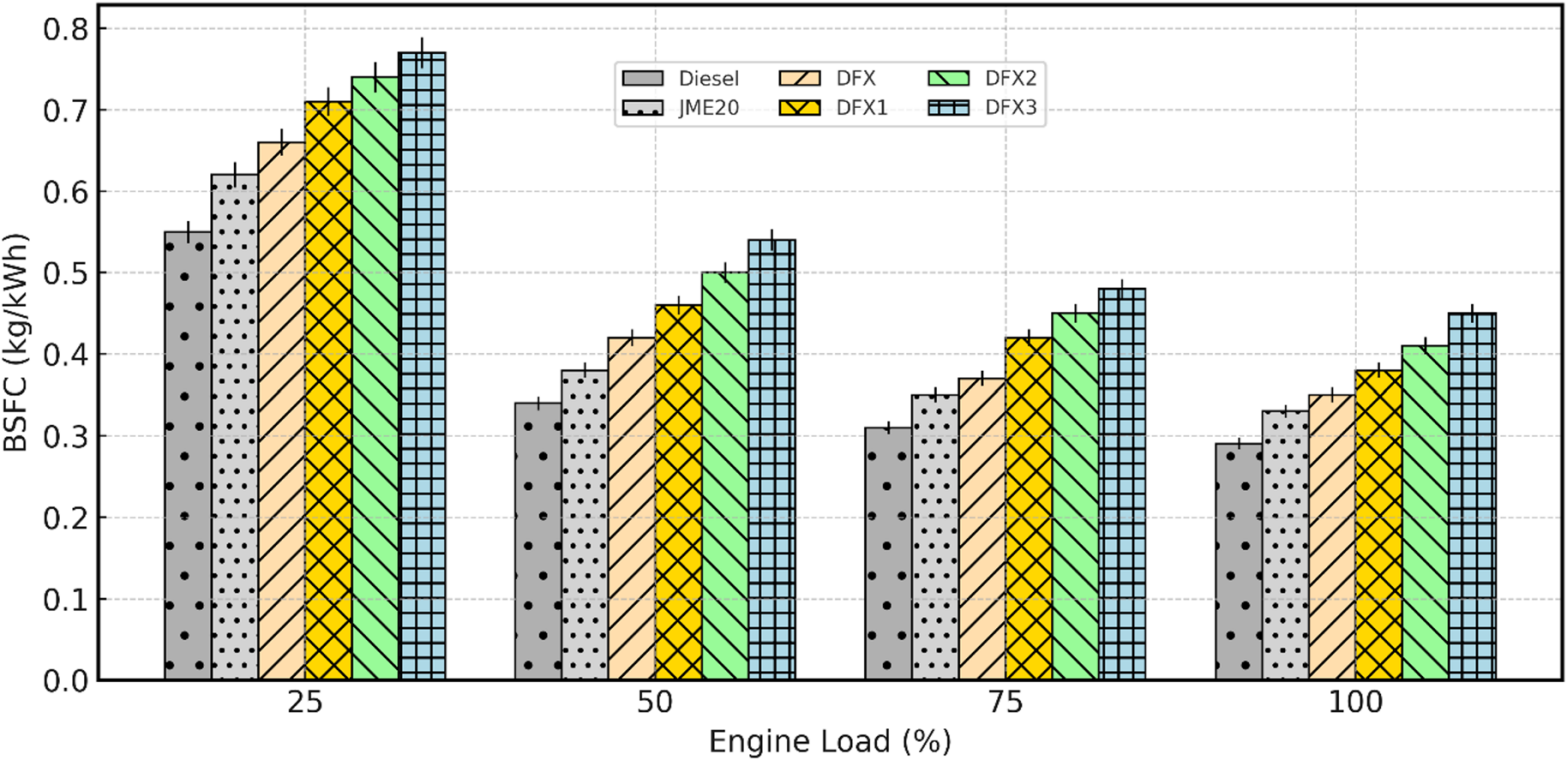
Variation of BSFC with respect to engine load.

BSFC is a measure of the fuel efficiency of an engine in terms of fuel consumption per unit of power produced. It is typically expressed in units like kilograms per kilowatt-hour (kg/kWh). The factors affecting BSFC are like engine design and type, operating temperature, type of fuel, and quality, etc. The variation of BSFC with engine load for diesel, JME20, and dual fuel operations is shown in Fig. 8.

For all test fuels, BSFC decreased as the load increased because of better combustion temperature and reduced relative heat losses. At full load, diesel achieved the minimum BSFC of about 0.29 kg/kW-hr, whereas B20 showed 0.33 kg/kW-hr, reflecting its lower heating value. BSFC for dual-fuel cases was consistently higher than that of diesel. At 50% load, DFX3 (16 lpm NH_3_) recorded 0.54 kg/kW-hr, approximately 58.8% higher than diesel. This increase is mainly due to NH_3_ having a lower energy density than diesel fuel. As the proportion of NH_3_ increases, the overall energy density of the fuel blend decreases. This can lead to higher BSFC because more fuel is needed to produce the same amount of work. The other reason is due to low calorific value and poor auto-ignition quality of NH_3_, which lengthens ID and displaces part of the intake air. At higher loads, elevated cylinder temperature improved NH_3_ oxidation, slightly reducing the BSFC relative to diesel. At full load, the BSFC values for diesel, DFX, DFX1, DFX2, and DFX3 are 0.29 kg/kW-hr, 0.35 kg/kW-hr, 0.38 kg/kW-hr, 0.41 kg/kW-hr, and 0.45 kg/kW-hr, respectively.

#### Exhaust gas temperature (EGT)

**Fig. 9:**
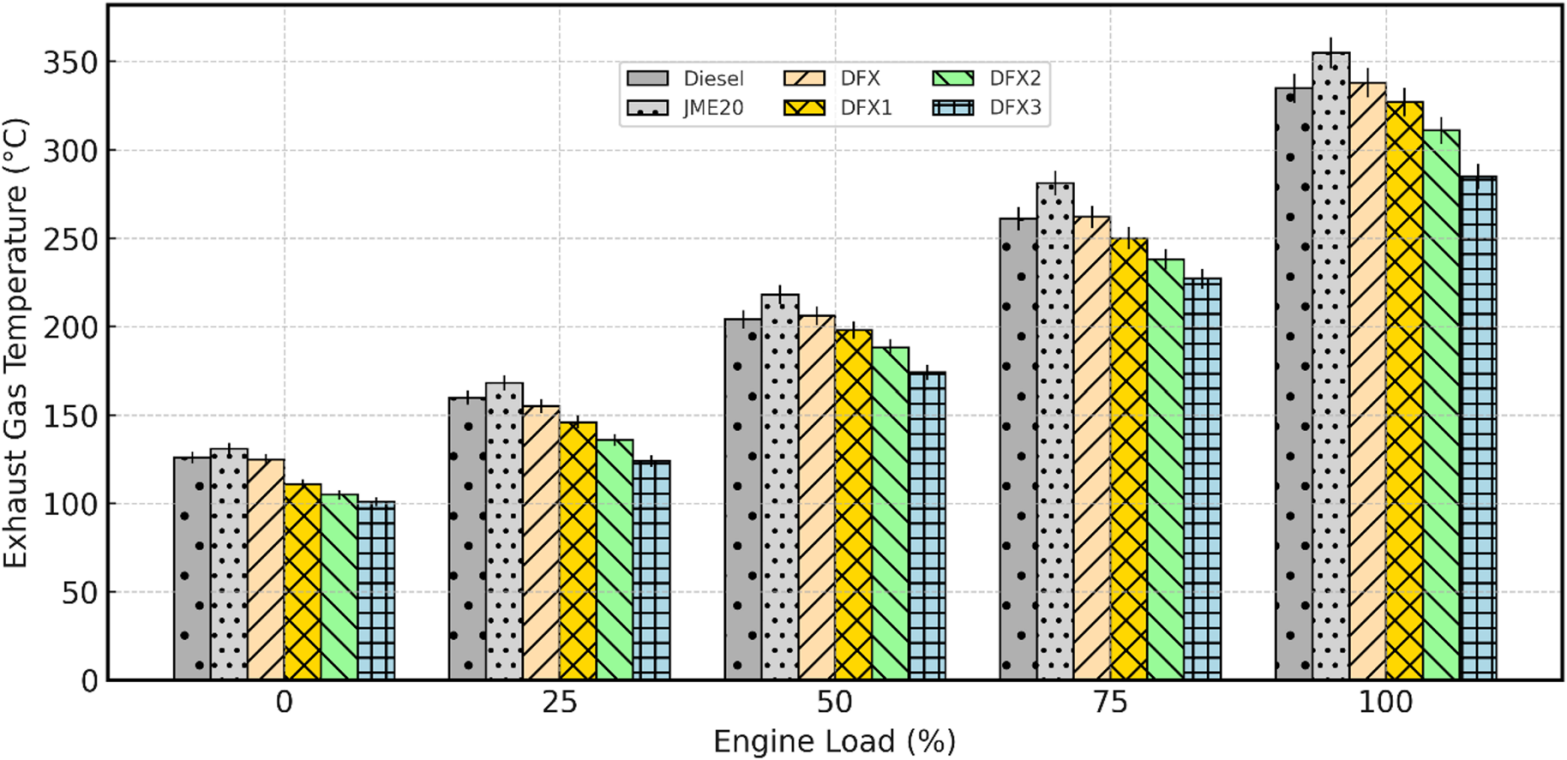
Variation of EGT with respect to engine load.

EGT in a diesel engine refers to the temperature of the gases exiting the combustion chamber and entering the exhaust system. It is a critical parameter because it reflects the efficiency of the combustion process and has implications for engine performance, emissions, and the durability of engine components. The trend of EGT with respect to engine load for diesel, B20, and dual fuel operations is shown in Fig. 9. For all test fuels, EGT steadily increased with load due to higher in-cylinder temperatures and greater energy release at higher engine loads. B20 exhibited the highest EGT at all loads, followed closely by diesel, owing to pre-bonded O_2_ molecules in biodiesel. At full load, the EGT of diesel and B20 is 335℃ and 355℃, respectively.

In dual-fuel operation with NH_3_ consistently resulted in slightly lower EGT compared to both diesel and B20 at all loads. This behavior is attributed to ammonia’s lower adiabatic flame temperature, slower burning rate, and its dilution of the intake charge, which together reduce peak gas temperatures. In other words, the combustion temperature decreases with higher NH_3_ content; thus, the temperature of the exhaust gases exiting the engine is also likely to decrease. The EGT values of DFX, DFX1, DFX2, and DFX3 are 338℃, 327℃, 311℃, and 285℃, respectively, at full load. It is worthwhile to note that the EGT for DFX3 is 14.9% lower than for pure diesel operation.

### Assessment of emission parameters

#### Carbon monoxide emissions

**Fig. 10:**
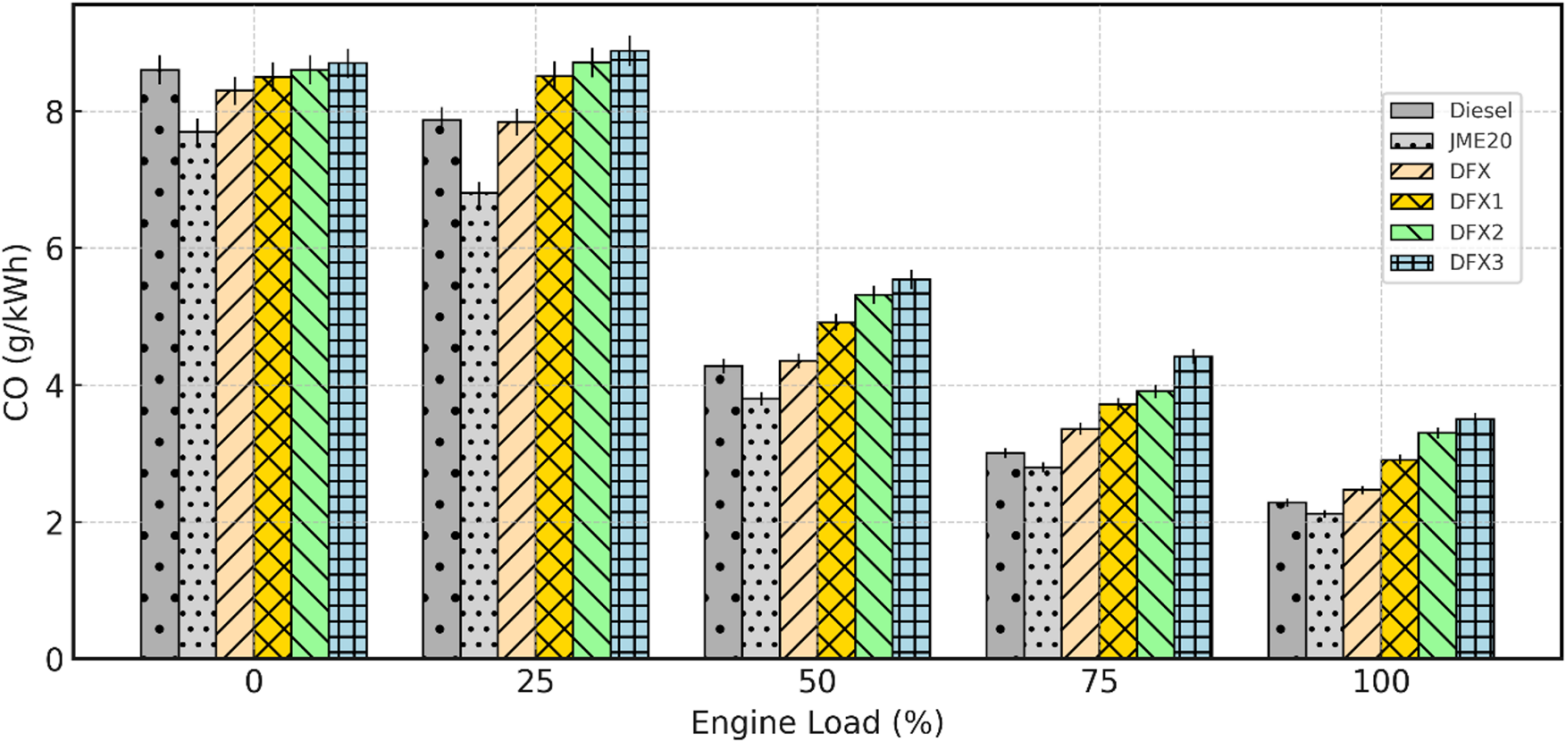
Variation of CO emission with respect to engine load.

CO emissions from a diesel engine refer to the amount of CO_2_ gas released into the atmosphere as a byproduct of the incomplete combustion of diesel fuel. CO is a colorless, odorless gas that is harmful to human health and contributes to air pollution. The prominent factors affecting CO emission are air-fuel ratio, fuel injection parameters, compression ratio, type of fuel/fuel properties, and O_2_ availability, etc. Figure 10 depicts the variation of CO emission with respect to engine load for diesel, B20, and dual-fuel operations. For all test fuels, CO emissions decreased steadily with increasing load, owing to high in-cylinder temperatures. Notably, B20 consistently emits lower CO levels than diesel at all loads; this improvement is attributed to the inherent O_2_ content of JME, which enhances oxidation of intermediate species.

In DFO, with an increase in the NH_3_ share, CO emissions tend to increase at all engine loading conditions. The rise in CO emission is a strong function of ignition-resistance and slower kinetics of NH_3_, which hinders the oxidation of CO species. NH_3_ induction into the intake port also reduces part of local O_2_ availability, forming a fuel-rich regions around the pilot spray. Hence, these regions experience an incomplete combustion, results to a rise in CO emissions^48^. Among all the DFOs, the DFX3 (16 lpm NH_3_) exhibited the highest CO levels. The CO emission values for diesel, B20, DFX, DFX1, DFX2 and DFX3 are 2.28 g/kWh, 2.12 g/kWh, 2.47 g/kWh, 2.91 g/kWh, 3.3 g/kWh, and 3.5 g/kWh respectively at full load. A percentage increase in CO emission of about 8.3%, 27.6%, 44.7% and 53.5% for DFX, DFX1, DFX2, and DFX3 operations, respectively, when compared to diesel at full load.

#### Unburnt hydrocarbon emissions

**Fig. 11:**
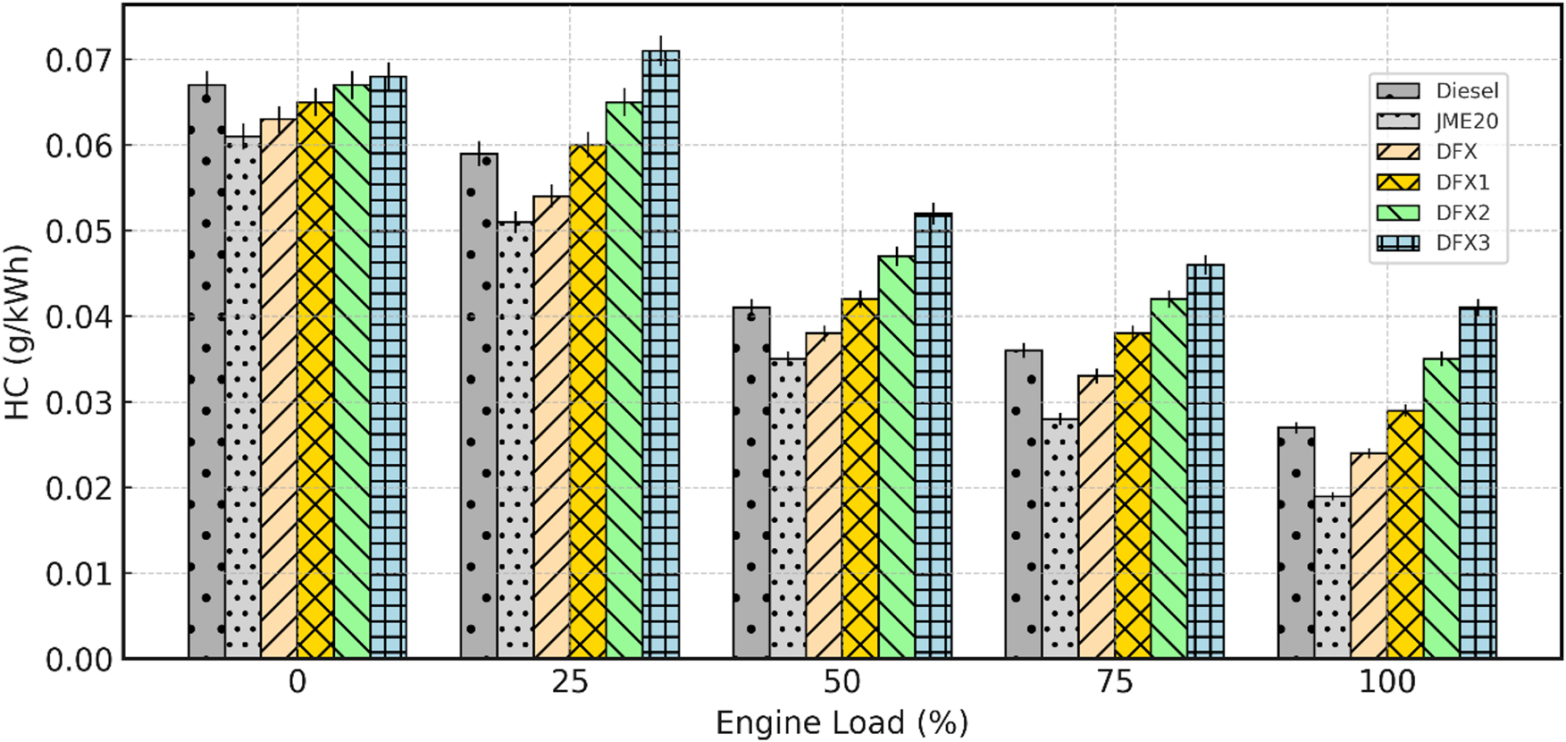
Variation of HC emission with respect to engine load.

HC emissions from a diesel engine refer to the release of unburned or partially burned hydrocarbons into the atmosphere. Hydrocarbons are organic compounds composed exclusively of carbon and hydrogen atoms, and their emission from diesel engines contributes to air pollution and can have adverse effects on human health and the environment. The parameters that affect HC emission are incomplete combustion of fuel, fuel properties, and fuel injection parameters, etc^49^. Figure 11 depicts the variation in CO emissions with engine load for diesel, B20, and dual-fuel operations. For all the test fuels, HC emission decline as load increases because of higher combustion temperature and turbulence enhance oxidation of unburned fuel. Diesel consistently exhibited the higher HC levels when compared to B20; this improvement in B20 is attributed to the oxygenated nature of B20, which promotes more complete combustion and hence reduces HC emissions.

The HC emissions values for diesel and B20 are 0.027 g/kWh and 0.019 g/kWh respectively at full load. In contrast, DFO exhibits higher HC emission when compared to diesel at all engine loading conditions. The increase in HC emissions is mainly due to key characteristics of NH_3_ such as lower flame temperature, slow flame speed, narrow flammability limits. This can be explained by the fact that, when NH_3_ replaces part of pilot fuel, the overall mixture temperature is reduced, preventing from complete oxidation. Moreover, the lower reactivity and narrow flammability limits also leads to a partial combustion, leaving a larger portion of crevice hydrocarbons unoxidized. The latter reason was documented by^46^ in the experimentation on a diesel engine using NH_3_ as a primary fuel. The HC emission values for DFX, DFX1, DFX2 and DFX3 are 0.024 g/kWh, 0.029 g/kWh, 0.035 g/kWh, 0.041 g/kWh respectively at full load.

#### Nitric oxide

**Fig. 12:**
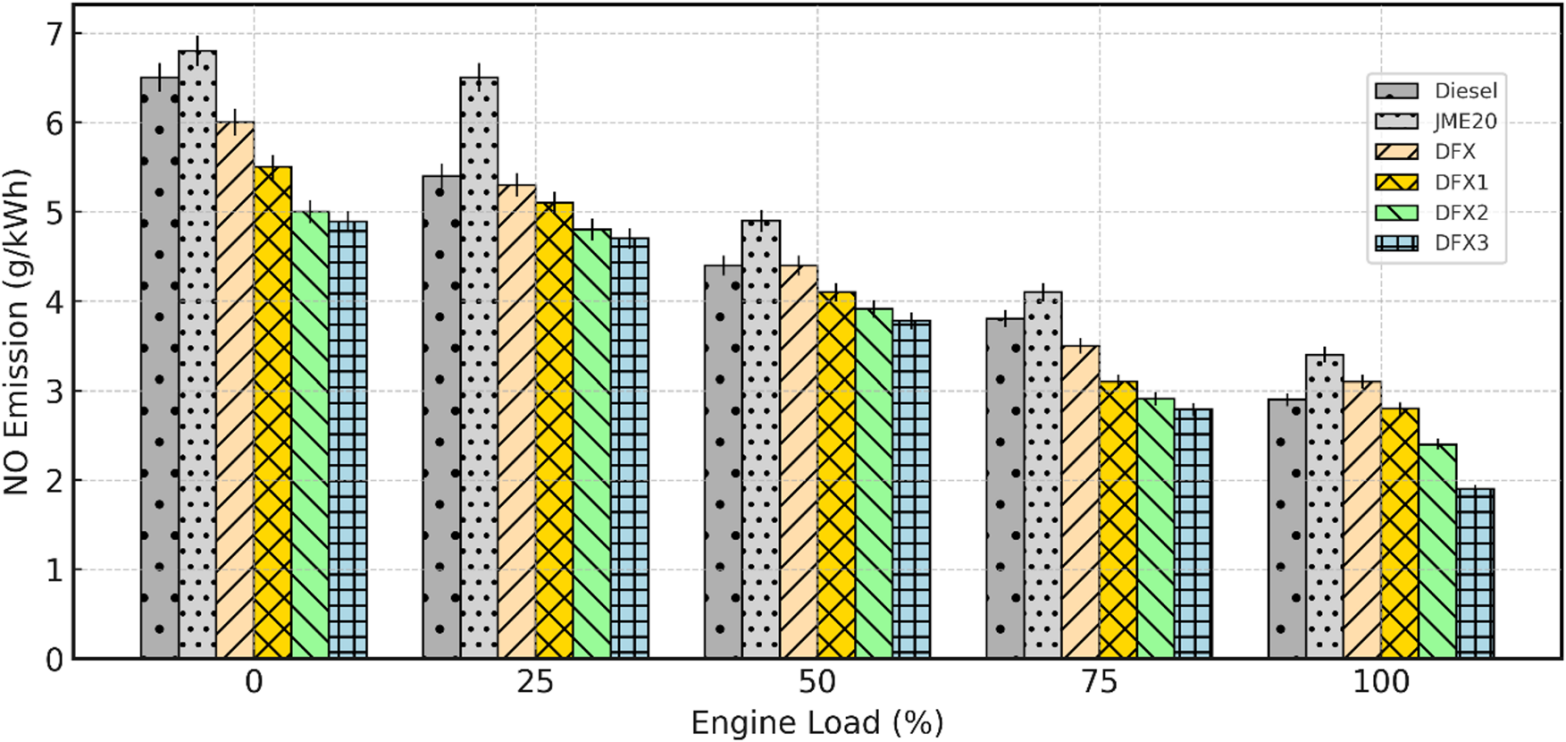
Variation of NO emission with respect to engine load.

NO formation in a CI engine can be greatly affected by cylinder temperature, O_2 _concentration, residence time. Figure 12 portrays the variation of NO emission with respect to engine load for diesel, B20, and dual-fuel operations. For all the test fuels, NO emissions steadily decline as load increases because the higher fuel-to-air ratio lowers the O_2_ availability per unit fuel and reduces peak flame temperature. But, B20 exhibits higher NO emission compared to diesel, reflecting its O_2_ content that enhances combustion temperature. The values of NO emissions for diesel and B20 are 2.9 g/kWh, 3.4 g/kWh respectively, at full load.

Though NH_3_ contains fuel-bound N_2_, NO emissions are found to be decreased with increase in NH_3_ flow rate. This trend is primarily due to NH_3_’s strong charge-dilution effect, which reduces the O_2_ percentage in the cylinder, thereby reduces the adiabatic flame temperature. Further, this trend can also be proven by the two dominant factors of thermal NO formation, suggested by Zeldovich mechanism. The slower reactivity and lower flame temperature of NH_3_ delayed the combustion process and shifted the heat release towards the expansion side. Because of this, the residence time and temperature are not sufficient for significant NO formation. The values of NO emission for diesel, B20, DFX, DFX1, DFX2 and DFX3 are 2.9 g/kWh, 3.4 g/kWh, 3.1 g/kWh, 2.8 g/kWh, 2.4 g/kWh, and 1.9 g/kWh respectively at full load.

#### Smoke opacity

**Fig. 13:**
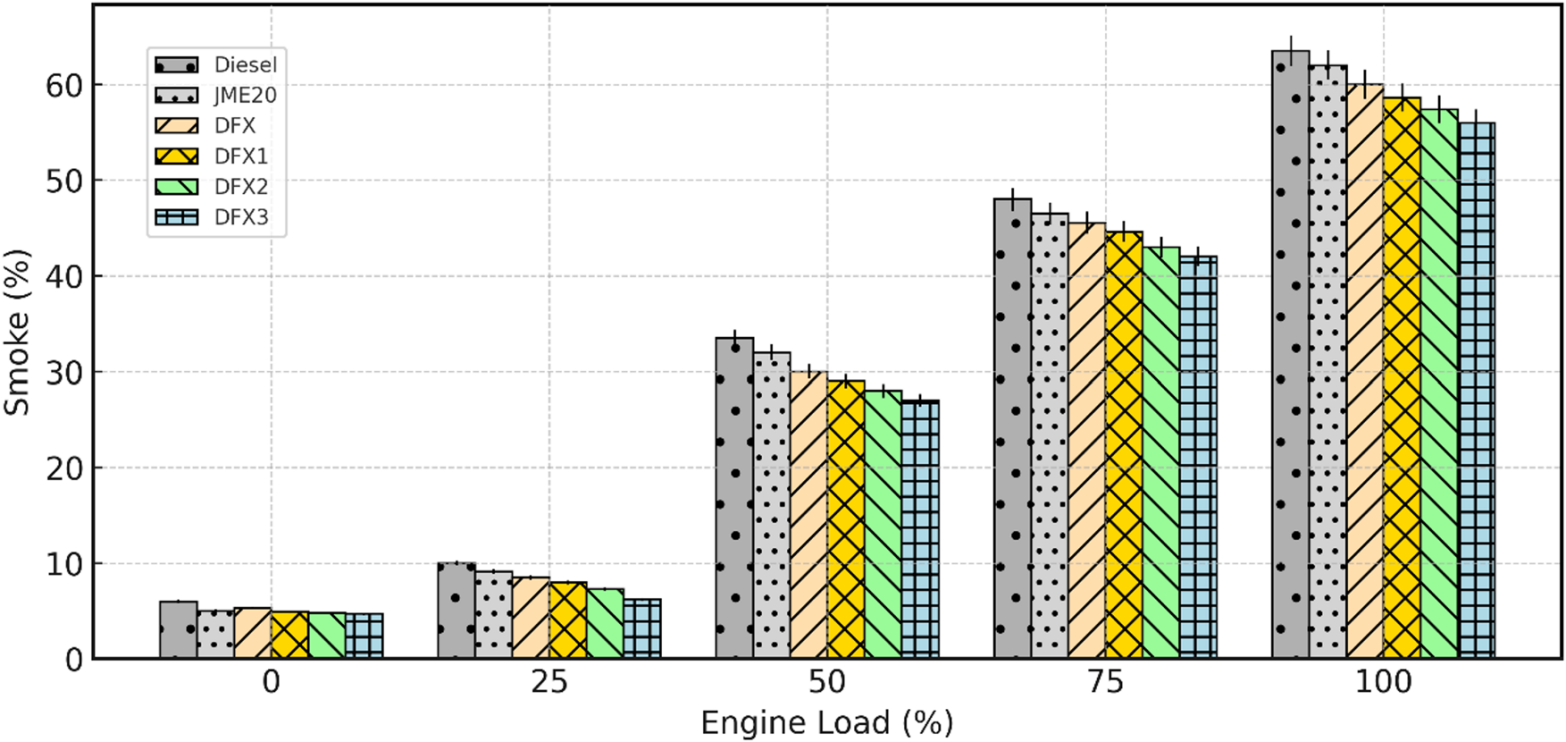
Variation of smoke emission with respect to engine load.

Smoke emission from a diesel engine refers to the visible particulate matter (PM) that is released into the atmosphere as a result of the incomplete combustion of fuel. The variation of smoke emission with respect to engine load for diesel, B20, and dual-fuel operations is shown in Fig. 13. Smoke emission increases steadily with an increase in load for all test fuels, because higher load enhances fuel quantity and rich zones, favouring soot formation. However, B20 exhibits slightly lower values owing to its inherent O_2_ content that improves oxidation of soot precursors.

In DFM, introducing NH_3_ further suppresses smoke emissions across all load range, with the maximum reduction observed at DFX3. This can be explained by the carbon-free nature of NH_3_, which lowers the amount of carbon available for soot nucleation and to better premixing at higher flow rates. In other words, due to the induction of gaseous NH_3_ by premixed combustion and reducing the amount of carbon in the mixture decreases the soot formation. The latter reason mentioned here is in good accordance with the reason mentioned by. Cheng et al.^46^. However, the further decrease in smoke emission from DFX2 to DFX3 is minimal, implying that most of the benefit is achieved at moderate induction levels.

## Conclusion

This study systematically evaluates the effect of NH_3_ induction on combustion, performance, and emission characteristics of a DI diesel engine operated on DFM using JME20 as a pilot fuel. The following key conclusions are drawn from this study;

- As the NH_3_ energy share increases, the λ value decreases, indicating a transition towards a richer air-fuel mixture. A maximum of 24.3% pilot fuel replacement was found for DFX3 at full load.
- With an increase in the flow rate of NH_3_, the peak pressure also increases. The lower combustion rate and slow flame speed of NH_3_ favours the ignition delay to prolong, which results in higher cylinder pressure in the premixed phase of combustion. PCP of DFX, DFX1, DFX2, and DFX3 are 61.8 bar, 55.41 bar, 56.56 bar, 58.72 bar, and 56.47 bar, occurred at 10.6°CAaTDC, 11.8°CAaTDC, 13.04°CAaTDC, and 15.42°CAaTDC, respectively, at full load.
- HRR also increases with an increase in the flow rate of NH_3_. Interestingly, at DFX3 operation (16 lpm), the HRR peak drops back to 51.7 J/°CA. This reduction is attributed to the excessive presence of NH_3_ in the combustion chamber, which suppresses the overall combustion rate.
- BTE decreases steadily with the increase in flow rate of NH_3_. A drop in BTE of about 6.6%, 10.9%, 14.7% and 25.7% for DFX, DFX1, DFX2, and DFX3, respectively, then that of diesel operation at full load.
- Both the ID and CD increase continuously with an increase in the flow rate of NH_3_. This is due to the presence of NH_3_, which has a low flame speed, high ignition resistance, and requires higher energy for sustained combustion.
- As the NH_3_ flow rate increases, it displaces part of the pilot fuel, and because of its low reactivity, the premixed NH_3_-air charge may not burn completely, leading to a reduction in the effective quantity of diesel undergoing combustion. Hence, both the CO and HC emissions increase. A percentage increase in CO & HC emission of about 44.7% and 29.6% for DFX2 test fuel, respectively, when compared to diesel at full load.
- NO emissions decreased progressively till DFX3, suggesting that the dilution and cooling effects of ammonia outweighed the additional fuel-bound nitrogen across the test range.

In summary, the test results indicate that NH_3_-JME20 operation is technically feasible for agricultural diesel engines, provided the NH_3_ replacement is carefully optimized. Among the test fuels, DFX2 (12 lpm) exhibited the most favorable combustion stability. Though NH_3_ flow rates resulted in longer delay and reduced efficiency, future work may also be explored at considering optimization of injection timing (to compensate for NH_3_’s slow reactivity), aftertreatment for CO/HC control, and real-time monitoring of NH_3_ slip. Additionally, the emission insights presented in this study provide qualitative environmental relevance. Future work will focus on quantitative environmental and enviro-economic assessment, incorporating CO–HC–NO–CO₂–PM impact factors, monetary damage cost functions, and ammonia-slip diagnostics based on methodologies reported in the literature^50–53^. This will extend the present combustion-emission baseline toward sustainability-level evaluation.

## Data Availability

The data will be made available upon request to the corresponding authors.

## Abbreviations

BTE: Brake Thermal Efficiency
BSFC: Brake Specific Fuel Consumption
CI: Compression Ignition
CD: Combustion Duration
CO: Carbon monoxide
CV: Calorific Value
CO_2_: Carbon Dioxide
DF: Dual Fuel
DFO: Dual Fuel Operation
GHG: Greenhouse Gas
HC: Hydrocarbon
HRR: Heat Release Rate
IC: Internal combustion
ID: Ignition Delay
JME: Jatropha Methyl Ester
lpm: Liter per minute
MCP: Maximum Cylinder Pressure
MHRR: Maximum Heat Release Rate
NO: Nitric oxide
NH₃: Ammonia
PCP: Peak Cylinder Pressure
SI: Spark-Ignition
SOI: Start of Injection
TDC: Top Dead Centre
Λ: Equivalence ratio
